# Cannulation Strategies in Type A Aortic Dissection: Overlooked Details and Novel Approaches

**DOI:** 10.7759/cureus.46821

**Published:** 2023-10-10

**Authors:** Indresh Yadav, Hanya Saifullah, Arun Kumar Mandal, Mohammed Khaleel I.KH. Almadhoun, Heba Mohamed Elsheikh Elabadi, Merceline Eugene, Majid Suleman, Hind Omer Bushra Himedan, FNU Fariha, Hanzala Ahmed, Muhammad Ali Muzammil, Giustino Varrassi, Satesh Kumar, Mahima Khatri, Mahir Elder, Tamam Mohamad

**Affiliations:** 1 Internal Medicine, Samar Hospital and Research Center Pvt. Ltd., Janakpur, NPL; 2 Internal Medicine, Community Based Medical College, Bangladesh, Mymensingh, BGD; 3 Medicine and Surgery, CMH Lahore Medical College and the Institute of Dentistry, Lahore, PAK; 4 Internal Medicine, Manipal College of Medical Sciences/Oda Foundation, Pokhara, NPL; 5 Medicine and Surgery, Mutah University, Al-Karak, JOR; 6 Medicine and Surgery, Faculty of Medicine and Health Sciences, Shendi University, Shendi, SDN; 7 Internal Medicine, Broward Health Medical Center, Florida, USA; 8 Cardiology, SHED Hospital, Karachi, PAK; 9 Medicine and Surgery, University of Gadarif, Tawawa, SDN; 10 Medicine and Surgery, Dow University of Health Sciences, Karachi, PAK; 11 Medicine and Surgery, Islamic International Medical College, Riphah International University, Karachi, PAK; 12 Internal Medicine, Dow University of Health Sciences, Karachi, PAK; 13 Pain Medicine, Paolo Procacci Foundation, Rome, ITA; 14 Medicine and Surgery, Shaheed Mohtarma Benazir Bhutto Medical University, Karachi, PAK; 15 Interventional Cardiology, Heart and Vascular Institute, Detroit, USA; 16 Cardiovascular, Wayne State University, Detroit, USA

**Keywords:** cardiopulmonary bypass, innovative techniques, novel approaches, cannulation, type a aortic dissection

## Abstract

Aortic dissection type A is a life-threatening condition that frequently necessitates surgical intervention. This review focuses on central aortic cannulation, arch branch vessel (ABV) cannulation, and proximal arch cannulation as key techniques during aortic surgery. It discusses innovative solutions for addressing these challenges. The review synthesizes findings from recent studies and emphasizes the significance of meticulous planning and execution of cannulation in aortic dissection repair. This review aims to contribute to the advancement of surgical practices and the enhancement of patient outcomes in the management of type A aortic dissection (AAD) by addressing these frequently overlooked details.

## Introduction and background

Stanford type A aortic dissection (AAD) is a life-threatening medical condition characterized by a tear in the inner layer of the aorta, the body's primary artery. This separation of aortic wall layers can result in the formation of a false lumen, allowing blood to travel between the layers and potentially causing organ damage, aneurysm formation, or rupture [1].

Aortic ascending dissection is a subset of aortic dissection that predominantly affects the ascending aorta. The condition manifests as chest pain, which is frequently described as severe and sudden in onset. Symptoms of hemodynamic instability, such as shock, syncope, and shortness of breath, can also result from decreased blood flow to vital organs [2].

Imaging techniques, such as computed tomography (CT) scans or magnetic resonance imaging (MRI), which permit visualization of the dissection site and aid in determining the extent of the rupture, are frequently utilized to aid in the diagnosis. Important for preventing life-threatening complications is prompt diagnosis and treatment. Surgical interventions to repair ruptured aortic layers and restore normal blood flow are included in treatment strategies [1].

A review of risk factors and outcomes associated with AAD emphasizes the necessity of comprehending the condition's predisposing factors. Among the factors that increase the risk of AAD are hypertension, connective tissue disorders, bicuspid aortic valves, and aortic aneurysms [3].

## Review

The significance of appropriate cannulation techniques during cardiopulmonary bypass (CPB)

Appropriate cannulation strategies are of utmost importance during CPB for aortic dissection type A (AAD). CPB requires rerouting blood flow away from the heart and lungs, relying on arterial cannulation to maintain circulation. Inappropriate cannulation may result in impaired perfusion, stroke, and even dissection extension.

Studies stress the importance of strategic cannulation in AAD repair, emphasizing the need for meticulous planning and execution. Due to their prospective benefits, techniques such as axillary cannulation and direct true lumen cannulation have gained attention [4][5]. Not only does proper cannulation assure the safety of CPB, but it also influences postoperative outcomes. By minimizing ischemic time and maximizing organ perfusion, well-conceived cannulation strategies play a crucial role in minimizing complications and optimizing patient recovery [6].

Cannulation techniques

Arterial Catheterization Throughout CPB

Arterial cannulation during CPB is a crucial surgical technique for AAD. The cannulation site selected is crucial for minimizing complications. Femoral cannulation, axillary artery cannulation, and innominate artery cannulation are common techniques. Cannulation of the femoral artery provides access for CPB. It can, however, increase the risk of aortic dissection extension. In contrast, axillary artery cannulation is considered safer for patients with AAD because it avoids the delicate aortic arch [6].

Recent research has investigated double arterial cannulation (DAC) strategies, incorporating right axillary artery and femoral artery cannulation, to improve surgical safety and efficacy in AAD patients. These strategies are intended to increase cerebral and systemic perfusion while decreasing the risk of complications [7].

Importance of Satisfying Perfusion Effects and Clinical Outcomes

During cannulation for AAD, it is crucial to achieve satisfactory perfusion effects and optimal clinical outcomes. A well-executed cannulation strategy assures a swift and sufficient perfusion flow, thereby minimizing organ malperfusion and complications. This has an immediate influence on patient safety and postoperative success. Strategies employing DAC, direct true lumen cannulation, and extra-anatomic revascularization are associated with better outcomes [7-9]. Putting an emphasis on cerebral protection during arch surgery increases the likelihood of successful outcomes [10]. Optimizing cannulation techniques has a significant impact on patient management and overall clinical outcomes [11].

Existing Literature on Cannulation Techniques in AAD and Their Limitations

In AAD surgery, cannulation strategies are crucial for optimal outcomes. To resolve the complexity of AAD, various approaches are utilized, each of which has its own limitations. Xia et al. [4] noted that, while various cannulation strategies, such as axillary artery and central aortic cannulation, are utilized, the choice is frequently influenced by patient-specific factors. Cannulation techniques rely heavily on anatomical knowledge. According to Pruna-Guillen et al., each strategy results in a distinct flow pattern within the aorta during CPB, which affects organ perfusion [12].

Rosinski et al. [3] examined the direct true lumen cannulation technique, discussing its benefits and drawbacks, which include advantages in certain cases but execution complications. Khan et al. [13] emphasized the significance of proximal arch cannulation in aortic dissection surgery. The article emphasizes that axillary artery cannulation is a common technique that requires careful consideration of its limitations [14]. While the literature recognizes the variety of strategies and their limitations, more research is required to refine the patient-specific selection process. To ensure the best possible outcomes for patients undergoing AAD surgery, surgeons must weigh the advantages and disadvantages of each approach.

Advantages and disadvantages of common cannulation techniques

Femoral Artery Angioplasty

In the event that aortic arch cannulation (AAC) fails in AAD procedures, femoral artery cannulation is utilized as an alternative. This method offers advantages such as accessibility and familiarity to surgeons, making cannula insertion and fixation straightforward. However, femoral artery cannulation has limitations, such as the potential risk of embolic events due to retrograde perfusion and reduced cerebral flow, which raises concerns regarding stroke and organ malperfusion. Notably, although femoral artery cannulation can provide a reliable alternative for CPB, careful consideration of its drawbacks and comparison with other cannulation strategies is necessary to ensure optimal outcomes in cases of AAD [15].

Ascending Aortic Catheterization

In AAD interventions, ascending AAC is a common technique. Direct access to the aorta, which permits efficient CPB and facilitates cooling of the ascending aorta, is one of the advantages of AAC. A disadvantage of AAC is that it does not entail clamping the ascending aorta during cooling, which may result in a loss of operating time and a prolonged procedure. In addition, retrograde blood flow and embolic events may occur during AAC, which may increase the risk of stroke and impair organ perfusion. In certain cases of acute aortic dissection, direct cannulation of the ascending aorta is regarded as a simple and safe alternative to other cannulation techniques [16].

Artery Cannulation of the Axillary

In AAD procedures, the axillary artery is cannulated. Advantages of axillary artery cannulation include a decreased risk of embolic events, the avoidance of retrograde flow, and improved organ perfusion compared to other techniques. When femoral arteries are compromised by dissection, it provides an alternative [17]. However, this method can be technically challenging and necessitate surgical expertise, leading to potential complications such as axillary artery injury and subclavian artery stenosis [18]. Despite this, axillary artery cannulation is regarded as advantageous in terms of cerebral and overall perfusion, making it a valuable surgical option for the treatment of AAD [19].

Comparative Analysis of Outcomes and Considerations for Each Strategy

Surgical procedures for AAD employ a variety of cannulation techniques, each with distinct outcomes and considerations. Direct cannulation of the ascending aorta provides simple access, but aortic injury is a risk. A femoral artery cannulation is an option when ascending aorta cannulation is difficult, but it can result in embolic events [12]. Cannulation of the axillary artery prevents retrograde blood flow and improves organ perfusion, but there are technical challenges and subclavian artery complications [19]. Dual arterial cannulation balances the benefits of axillary and femoral cannulation, while central cannulation is gaining favor [20]. A study of the STS database and two meta-analyses found an increased risk of stroke and short-term mortality with femoral cannulation compared to axillary cannulation [21-23]. However, femoral cannulation is more expedient and is regarded as the primary arterial site in patients with hemodynamic instability requiring immediate cannulation or anatomical characteristics preventing axillary cannulation. Each method has distinct effects on patient survival, cerebral perfusion, and malperfusion hazards [11,24]. The significance of individualized decision-making in AAD surgery is highlighted by the fact that the choice depends on anatomical considerations, surgeon expertise, and patient factors.

**Figure 1:**
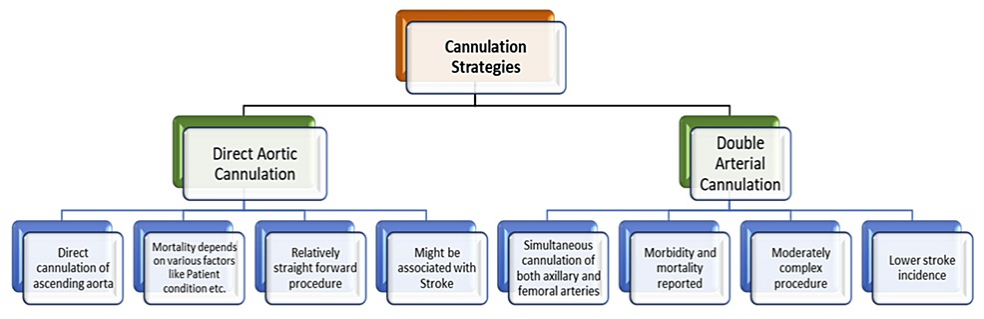
Comparing direct aortic cannulation and double arterial cannulation.

Figure 1 illustrates the distinctions between direct aortic cannulation and DAC in terms of advantages and disadvantages [25-28].

Retrospective studies on cannulation techniques

Evaluation of Data from Retrospective Investigations

Cannulation strategies for AAD have evolved to improve patient outcomes and surgical techniques. Optimizing cerebral and visceral perfusion while minimizing complications is a contemporary trend. Recent studies emphasize the significance of selecting cannulation strategies that offer superior cerebral protection and reduce the risk of malperfusion [25]. Cannulation strategies for AAD have been significantly enhanced by retrospective studies. The significance of these strategies and their effect on patient outcomes were highlighted in a comprehensive analysis. According to the study, each cannulation has advantages and disadvantages that must be weighed thoroughly prior to surgery. DAC is recommended because it can provide a model of double and combined blood perfusion. It should be noted that none of the cannulation strategies are ideal for all AADs. Preoperative and intraoperative malperfusion, particularly CPB-related malperfusion of the brain or cannulation-related malperfusion, must be identified using various CTA techniques, arterial pressure change, transesophageal echocardiography (TEE), TCD, and cerebral oximetry monitoring [26].

A second Chinese retrospective study examined the management strategy for AAD and emphasized the need for effective strategies to treat this fatal condition [29]. In addition, the 2022 ACC/AHA for Aortic Disease Diagnosis and Management emphasizes the significance of evidence-based decisions and proper consideration of cannulation strategies to enhance patient care [11]. In patients with cerebral malperfusion, surgery enhances survival; in patients with acute AAD and acute stroke, mortality rates are 25%-27% versus 76%, respectively, for surgical versus medical management [30,31]. Several series of acute AAD surgeries have reported direct aortic cannulation utilizing the Seldinger technique guided by TEE. This technique has the advantage of rapidly establishing CPB with true lumen flow when performed accurately. However, when the patient is stable, the technique's safety in comparison to axillary cannulation is questionable [26], as stroke rates with this technique can reach 20% in some series. Rosinski et al. [3] discovered that patients undergoing direct aortic cannulation were more hemodynamically unstable and had longer repairs; however, even in multivariable logistic regression, it was linked to an increased risk of stroke (OR=2.3; 95% CI=1.05-5.1) [32]. In cases of protracted circulatory arrest, cerebral perfusion via a route other than axillary perfusion will be required. Cannulation of the innominate artery provides access for antegrade cerebral perfusion and appears to be safe in acute AAD [33-35]. The comparison of single axillary cannulation to dual arterial cannulation has shown that single axillary artery cannulation was as effective as dual arterial cannulation in complex arch correction for acute aortic dissection. When axillary artery cannulation failed to attain an adequate flow volume or when lower limb malperfusion was present, femoral artery cannulation was necessary [36].

Cannulation and its important factors

Frequently Neglected Factors in Cannulation Decision-Making

Cannulation decisions in AADs are crucial for achieving successful surgical outcomes, yet numerous factors are frequently disregarded. Each cannulation technique interacts uniquely with the aorta's anatomy and blood flow patterns [37]. Anatomical abnormalities, such as tortuosity or calcification, may impact the accessibility and safety of the cannulation site. In addition, the surgeon's skill and familiarity with the selected cannulation technique are crucial. The success of cannulation and the efficacy of an operation can be affected by variables such as the surgeon's familiarity with and skill with a particular technique. The importance of patient-specific characteristics cannot be overstated. Preexisting conditions, such as ankylosing spondylitis and hypertension, can impact the decision to catheterize [37,38].

Recent technological advancements and innovations, such as simplified surgical techniques, also merit consideration. This can lead to better results for institutions with limited resources [39]. It is essential to balance the situation's urgency with the need for meticulous planning. Immediate management of acute aortic dissection requires prompt decision-making, but the choice of cannulation technique should not compromise patient safety or long-term outcomes.

Effect of These Neglected Particulars on Perfusion and Results

In AAD, the efficacy of cannulation strategies has a significant impact on perfusion and surgical outcomes. Inadequate cerebral and visceral perfusion can have a negative effect on patient outcomes if crucial cannulation considerations are neglected. Cannulation techniques, flow patterns, and cannulation site selection have a direct impact on perfusion and complications during CPB [39].

Novel strategies and methods

Introduction to Innovative and Emergent Cannulation Techniques

Emerging and novel cannulation techniques for AAD are revolutionizing surgical interventions for this life-threatening condition. These approaches seek to enhance cannulation strategies, optimize perfusion, and improve patient outcomes, as detailed in numerous studies. Cannulation has traditionally been a crucial component of aortic surgery, influencing cerebral and visceral perfusion. However, novel techniques, such as direct true lumen cannulation and dual arterial cannulation, are garnering attention for their potential to address difficulties associated with conventional methods. In addition to ensuring optimal perfusion, these techniques seek to minimize the risk of complications and malperfusion [38].

Case Studies and Successful Examples of Novel Approaches

Several effective novel cannulation strategies have been used to improve surgical outcomes in cases of AAD:

True lumen direct cannulation: Direct true lumen cannulation (TLC) is being investigated as a potential alternative to conventional cannulation in certain cases. It is applicable in complex situations. This involves carefully inserting the cannula into the true lumen, as opposed to the false lumen, in order to ensure appropriate perfusion [39].

Arterial cannulation at Botallo's ligament: Direct arterial cannulation of the actual lumen of the dissected aorta at the level of Botallo's ligament has been utilized successfully [40].

Strategy for combined femoral and axillary perfusion: Patients with AAD have a better prognosis if both the femoral and axillary arteries are cannulated for perfusion [41].

Supra-aortic cannulation: Supra-aortic cannulation techniques can avoid manipulations on fragile arch arteries, thereby improving patient safety during perfusion cannula insertion. Initial axillary cannulation systematic initial axillary cannulation for acute type A dissection repair has been shown to be safe and effective [42].

Cannulation of the proximal arch: Clinical implementation of proximal arch cannulation has demonstrated its importance in surgical treatment, especially in cases of acute DeBakey type I aortic dissection [14].

These strategies highlight advances in aortic dissection repair that are tailored to the complexity of individual cases and contribute to better patient outcomes.

Possible Advantages and Difficulties of Implementing Innovative Techniques

Implementing novel cannulation techniques in the surgical treatment of AAD presents both prospective benefits and obstacles. Innovative techniques, such as direct true lumen cannulation and the frozen elephant trunk (FET) method, are designed to enhance perfusion and repair. Direct cannulation of the true lumen reduces the risk of false lumen perfusion, thereby enhancing organ preservation. The FET technique facilitates proximal repair extension, which contributes to enhanced outcomes [43]. These techniques have the potential to improve surgical success, thereby minimizing complications and subsequent interventions. However, obstacles include mastering these sophisticated techniques to prevent complications and ensuring that they are appropriate for each patient's unique anatomy. Failure to correctly identify and resolve cannulation issues can jeopardize the success of a surgical procedure. Additionally, the adoption of novel methods necessitates extensive training, which may initially lengthen surgical times [44]. Surgeons must weigh the prospective benefits against the learning curve and the need for meticulous preoperative patient selection. The 2022 ACC/AHA Guideline for Aortic Disease Diagnosis and Management provides clinicians with guidance for overcoming these obstacles [11].

Tables 1-2 provide a summary of the advantages and disadvantages of various cannulation strategies [45-49].

**Table 1:** Different cannulation sites and techniques used in type A aortic dissection surgeries.

Cannulation Site/Technique	Advantages	Drawbacks
Subclavian/Axillary Artery	Appropriate for cerebral perfusion, evades an aortic cannulation	Unfavorable anatomy, risk of mal perfusion [5]
Femoral Cannulation	Used commonly, easily accessible	Inadequate cerebral perfusion [42]
Samurai Cannulation (Direct True-Lumen)	Avoids false lumen, improves cerebral perfusion	Technical challenges, limited applicability [46,47]
Aorta Echocardiography-guided Cannulation	Better visualization, precise placement	Skill-intensive, requires specialized equipment [48,49]
Multi-site Perfusion Strategy	Addresses mal perfusion and provides cerebral perfusion	Complexity, coordination challenges [50]

**Table 2:** Comparison of different cannulation strategies and their advantages and disadvantages.

Cannulation Strategy	Cannulation Site	Advantages	Disadvantages	Associated Outcomes
Axillary artery Cannulation	Axillary artery	Decreases the risk of stroke	Potential of nerve injury	Decreased cerebral embolism [4,5]
		Easier access in places where anatomy is difficult	Flow dynamics are limited	Lesser incidence of complications related to neurology [8]
Aortic Cannulation	Ascending aorta	Simple and already known technique	Might dislodge atheromatous debris	Established method with better outcomes [12]
		Balanced flow distribution	Risk of aortic dissection propagation	Suitable for standard anatomy [20]
Arch Branch Vessel Cannulation	Brachiocephalic vessels	Allows antegrade cerebral perfusion	Complex, requires skillful navigation	Better cerebral protection [45]
		Maintains brain oxygenation during bypass	Potential vessel damage	Preferred in patients with cerebral mal-perfusion
Direct True Lumen Cannulation	True lumen of the dissected aorta	Avoids false lumen perfusion	Technically challenging	Prevents retrograde dissection extension and mal-perfusion
		Decreases the risk of false lumen pressurization	Requires meticulous dissection	Good for retrograde dissection, improves outcomes

Significance of patient-specific cannulation techniques

Emphasizing the Importance of Individualized Cannulation Strategies

Management of AAD requires individualized cannulation techniques. Several factors require a customized approach:

Patient variability: The anatomy and dissection extent of each patient vary. By adapting to individual differences, personalized cannulation reduces the risk of complications such as stroke, organ malperfusion, and hemorrhage.

Maximizing cerebral perfusion: Time is crucial for minimizing ischemia [50]. Personalized strategies examine whether cannulation of the central aorta or femoral artery is more appropriate, thereby preventing brain damage due to inadequate blood flow [51].

Avoiding complexity: Surgery for aortic dissection is complex. Personalization decreases the likelihood of cannulation failure, which can result in extended operative durations and increased morbidity [51].

Guidelines and studies: The 2022 ACC/AHA Guideline for Aortic Disease Diagnosis and Management emphasizes the necessity of evidence-based strategies for the management of aortic disease [11]. Recent research in this field reveals that without a personalized approach, crucial details may be neglected.

The strategy for cannulation should be determined by the patient's anatomy, the extent of dissection, and the surgeon's skill. These factors must be carefully considered to ensure successful surgical outcomes and minimize complications during CPB for AAD. In AAD surgery, personalized cannulation strategies are essential for improving patient outcomes and minimizing complications. They take into account the unique characteristics of each patient's condition, ensuring effective blood flow and minimizing the risk of adverse events [52].

Function of Preoperative Imaging and Evaluation

Imaging and evaluation prior to AAD surgery play a crucial role in determining the optimal cannulation strategy. High-resolution techniques, such as CT, MRI, and TEE provide crucial information regarding the location, extent, and involvement of critical structures in the dissection [52].

These findings influence the choice of cannulation site to ensure cerebral and visceral perfusion during surgery. The preoperative evaluation of patient-specific variables such as aortic root dimensions and arch anatomy guides the decision between central aortic and femoral cannulation. In addition, preoperative imaging facilitates the identification of complicating factors, such as pericardial hematomas, which may affect cannulation and surgical approaches [53].

Specific cannulation methods for better perfusion

Anterograde Brain Blood Flow

In AAD surgery, anterograde cerebral perfusion is essential to ensure adequate oxygenation of the brain's blood supply. Diverse cannulation techniques have been investigated for this purpose. Typically, the axillary artery and innominate artery are deemed cannulation sites. The decision between these techniques is influenced by patient anatomy and surgeon preference.

Both axillary and innominate artery cannulation techniques provide effective anterograde cerebral perfusion, according to research. Studies such as "cannulation strategies in AAD" by Xia et al. [4] emphasize the significance of selecting an appropriate cannulation strategy to improve cerebral perfusion. In addition, Harky et al. and Lin et al. discussed the non-inferiority of innominate artery cannulation compared to axillary artery cannulation for ACP, which contributes to the comprehension of optimum cannulation techniques [34,54].

Retrograde Inferior Vena Cava Perfusion (RIVP)

RIVP is performed in AAD surgery to provide oxygenated blood to vital organs while temporarily halting the heart. Various strategies for enhancing RIVP in aortic surgery have been the subject of studies. Xia et al. [4] addressed cannulation strategies for AAD, focusing on the combination of RIVP with ACP to improve outcomes. To safeguard organs during surgery, Ciu et al. proposed continuous retrograde perfusion through the inferior and superior vena cavae simultaneously [55,56]. In order to optimize RIVP during aortic arch replacement surgery, Sarkar et al. describe a technique involving the tethering of both the superior and inferior vena cavae to the cannula [57].

Right Carotid Artery Perfusion

Enhancing reperfusion via the right carotid artery during surgery for AAD can be crucial for preserving cerebral perfusion. To accomplish this, specific cannulation techniques are employed. The right common carotid artery is cannulated immediately prior to CPB, notably in cases of brain malperfusion. While repairing the aorta, these techniques are utilized to ensure adequate blood flow to the brain. Studies such as "cannulation strategies in AAD" highlight the significance of arterial cannulation to facilitate reperfusion via the right carotid artery and support cerebral circulation during surgery. Despite the fact that the literature emphasizes the significance of cannulation strategies for reperfusion, specific technical details may vary depending on the characteristics of the individual patient [58].

Monitoring and evaluation throughout cannulation

Parameters to be Evaluated Throughout Cannulation and CPB

Critical parameters must be scrupulously monitored during cannulation and CPB in AAD to ensure patient safety and optimal outcomes. These factors consist of the following:

Continual monitoring of blood flow rates is necessary to ensure adequate oxygenation and perfusion of vital organs while minimizing the risk of malperfusion.

Blood pressure: Close monitoring of blood pressure helps maintain stable hemodynamics and prevents hypotension or hypertension-related complications.

Oxygen saturation: Monitoring oxygen saturation levels ensures adequate oxygen delivery to tissues and aids in detecting oxygenation-related problems.

Monitoring and moderating the body's temperature is essential for preventing hypothermia, which can negatively impact coagulation and organ function. Regular assessment of arterial blood gases provides insight into acid-base balance and respiratory function, guiding intervention.

End-organ perfusion: Continuous monitoring of end-organ function, such as renal and cerebral perfusion, is required to detect any indications of malperfusion and allow for prompt intervention.

Cannula position and function: It is essential to monitor the position and function of cannulas to prevent malpositioning and thrombus formation and to ensure effective perfusion. Regular surveillance of coagulation parameters aids in the prevention of bleeding or clotting complications [59].

Rapid Diagnosis and Treatment of Complications

In AAD, early detection and prompt management of complications during cannulation are crucial for patient outcomes. Recognition and intervention in a timely manner can prevent further deterioration and increase survival rates. Intensive monitoring of vital signs, such as blood pressure, heart rate, and oxygen saturation, is essential for detecting any indications of malperfusion or hemodynamic instability. Diagnostic imaging techniques, such as CT scans and TEE, play a crucial role in visualizing the dissection's extent and identifying potential complications, such as thrombosis or organ malperfusion [60].

Clinical consequences of cannulation techniques

Relationship Between Catheterization and Postoperative Complications

Various cannulation techniques carry distinct complications. Stenosis is a prevalent complication of arteriovenous fistulas, regardless of the cannulation technique [61]. End-organ dysfunction, coagulopathy, hemodilution, hemorrhage, and infection are potential complications of minimally invasive procedures involving CPB [62]. Each cannulation strategy (femoral, axillary, or central aortic) for AAD is associated with distinct advantages and potential complications, including malperfusion and aortic dissection extension. Risks associated with peripheral IV device management include infection, phlebitis, and accidental arterial cannulation [63-65]. These complications highlight the importance of selecting the appropriate cannulation technique, possessing the necessary skills, and considering patient-specific factors in order to minimize risks and maximize success. These complications are outlined in Table 3 below.

**Table 3:** Different cannulation techniques and complications associated with them.

Cannulation Techniques	Complications
Arteriovenous Fistula	1. AVF Complications 2. Thrombosis 3. Mal-perfusion [4,8,11]
Aortic Dissection Repair	1. Malperfusion 2. Distal re-entry 3. Embolization and access issues [20,45]
Axillary/Dual Arterial Cannulation	1. Malperfusion-related complications 2. Renal Failure 3. Intestinal ischemia [61]
Acute Type B Aortic Dissection	1. Malperfusion 2. Rupture [66,67]

Long-Term Effects on the Quality of Life of the Patient

The long-term effects of cannulation in AAD can have a negative impact on a patient's quality of life. Cannulation strategies, such as direct true lumen cannulation (DTLC), have been used to restore perfusion; however, they carry risks, such as further dissection or perforation of the aortic wall at the insertion site, which may negatively impact patients' long-term outcomes [68]. Despite being life-saving, aortic dissection surgeries can reduce the quality of life due to factors, such as surgical complications, postoperative discomfort, and potential permanent neurological deficits [64]. Long-term survival studies of patients with acute aortic dissections reveal obstacles that impact their overall quality of life. Consequently, although cannulation strategies and aortic dissection surgeries are essential for survival, they can have a long-term impact on patients' quality of life, necessitating careful consideration of the surgical approach and management for optimal outcomes [67].

Future directions and needs for research

Further Inquiry and Scope of Investigation

Several aspects of cannulation strategies for AAD could be explored further. It is crucial to investigate the efficacy and safety of various cannulation techniques in terms of long-term outcomes and quality of life. Exploring innovations in minimally invasive techniques and evaluating their effect on patient recovery could improve postoperative outcomes. In addition, refining perfusion strategies to mitigate complications such as cerebral and visceral ischemia and comprehending their effects on patient prognosis merits consideration [29,60]. Investigating the effect of patient-specific anatomical variations on cannulation efficacy and patient outcomes could inform the development of individualized interventions. In addition, it would be advantageous to incorporate computational simulations and advanced imaging techniques to optimize cannulation strategies and predict potential complications [60]. Longitudinal investigations comparing multiple cannulation techniques in larger patient cohorts can provide valuable information regarding clinical outcomes and quality of life [67]. Refining the management of AAD necessitates a comprehensive investigation into cannulation strategies, considering patient-centered outcomes and utilizing cutting-edge technologies.

Implementing Technological Advances in Cannulation Techniques

The incorporation of technological advances into cannulation techniques for AAD has the potential to improve patient outcomes. High-resolution imaging techniques, such as 3D angiography and intraoperative echocardiography, permit precise evaluation of aortic anatomy, thereby facilitating cannulation site selection and minimizing complications [36]. Simulation tools and computational modeling allow for preoperative planning that optimizes cannula placement for minimal impact on blood flow dynamics. Robotics and image-guided navigation systems provide precise cannula insertion and reduce the risk of vessel injury. During surgery, sophisticated perfusion monitoring devices can continuously assess tissue oxygenation and perfusion, facilitating in-the-moment decision-making. The incorporation of these innovations into surgical planning and execution can reduce complications, enhance perfusion, and optimize outcomes for patients with AAD [61].

Possibility of Randomized Controlled Trials (RCTs) in Cannulation Techniques

Significant potential exists for RCTs involving cannulation approaches for AAD. The rigorous evaluation of various cannulation strategies made possible by RCTs provides high-quality evidence to guide clinical practice. Currently, clinical outcomes are reported via comparative analyses, but prospective RCTs are essential for evaluating the safety, efficacy, and impact of various cannulation techniques. Taking into account factors such as perfusion quality, complication rates, and patient outcomes, these trials can help determine the optimal cannulation technique. Ongoing trials, such as the "Scandinavian trial of uncomplicated aortic dissection therapy," are a step in this direction, with the goal of providing valuable insights regarding effective treatment strategies [63]. Multicenter trials, such as those comparing axillary artery cannulation to innominate artery cannulation, provide a platform for evaluating innovative techniques. RCTs have the potential to shape evidence-based guidelines, refine surgical practices, and enhance patient outcomes for AAD [54].

## Conclusions

In conclusion, the review of cannulation strategies in AAD emphasizes the significance of seemingly insignificant surgical planning details. It highlights the importance of identifying and addressing unsuccessful cannulations using novel insights. The main findings of the review emphasize the necessity of a comprehensive evaluation to optimize cannulation decisions and ensure the highest quality of care. Surgical outcomes are highly dependent on the selected approach, necessitating precision and well-informed decisions. Continuous research is essential for refining and perfecting these strategies and enhancing patient outcomes. These studies have the potential to revolutionize surgical practices and improve patient care in cases of AAD by perpetually pushing the boundaries.
